# Effects of industrial heat treatments on the kinetics of inactivation of antimicrobial bovine milk xanthine oxidase

**DOI:** 10.1038/s41538-019-0046-8

**Published:** 2019-08-02

**Authors:** Gulustan Ozturk, J. Bruce German, Juliana M. L. N. de Moura Bell

**Affiliations:** 1 Department of Food Science and Technology, University of California, Davis, One Shields Avenue, Davis, CA 95616 USA; 2 Foods for Health Institute, University of California, Davis, One Shields Avenue, Davis, CA 95616 USA; 3 Biological and Agricultural Engineering, Davis, One Shields Avenue, Davis, CA 95616 USA

**Keywords:** Oxidoreductases, Oxidoreductases

## Abstract

Milk is a source of antimicrobial systems such as xanthine oxidoreductase, which has been proposed to modulate the oral and gut microbiota of infants. Heat treatments are applied to milk to ensure its microbial safety, however, the effects of heat on this antimicrobial enzyme are not known. The effects of batch pasteurization (BP), high-temperature short time (HTST), and ultra high temperature (UHT) on kinetics of inactivation of xanthine oxidase and its antimicrobial properties were determined. Xanthine oxidase activity was preserved by HTST (100%). Partial (8%) and nearly complete (95%) enzyme inactivation were observed for BP and UHT milks, respectively. *K*_m_ values of 100 μM and *V*_max_ values of 6.85, 5.12, 6.31, and 0.40 μmol/min/mg were determined for xanthine oxidase in raw, BP, HTST, and UHT milks, respectively. These results demonstrate that xanthine oxidase maintains apparent affinity and activity for its substrate when milk is treated by BP and HTST and yet the enzyme is inactivated with UHT. To investigate heat treatment-induced alterations in the biological activity of xanthine oxidase, heat treated milks were compared to raw milk for their ability to inhibit the growth of *S. aureus*. Raw, BP, and HTST milk xanthine oxidase efficiently inhibited *S. aureus* growth. However, these antibacterial properties were lost when milk was subjected to UHT. These results demonstrate that HTST and BP preserves bovine milk xanthine oxidase activity compared with UHT and that, the judicious selection of thermal treatments could be exploited to preserve the antimicrobial properties of bovine milk.

## Introduction

**Fig. 1:**
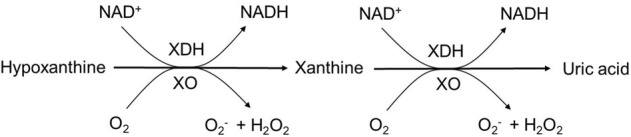
Xanthine oxidase (XO) and xanthine dehydrogenase (XD) catalyzes two step reaction. Xanthine oxidoreductase (XOR) exists in two interconvertible forms, NAD+-dependent dehydrogenase, (XDH), and oxygen-dependent oxidase (XO). XDH favors the cofactor NAD+ as its primary electron acceptor, yet XO is unable to bind NAD+ and uses O_2_ as its electron acceptor. Both forms catalyze the conversion of hypoxanthine into xanthine and then further into uric acid (UA). The figure is adapted with permission from ref. ^8^, copyright (Elsevier, 2003)

Milk is a complex fluid that evolved to provide complete postnatal nutrition for mammalian infants.^1^ Research is revealing that milk also acts to provide a selective microenvironment for the development of the oral and gut microbiota in the neonate.^2–5^ Milk both feeds and prevents microbial growth. Milk is a source of enzyme, such as xanthine oxidoreductase, that recent research has shown to produce anti-microbial metabolites, notably H_2_O_2_. These metabolites have been interpreted to modulate both the oral and gut microbiota.^5^ Xanthine oxidoreductase is widely distributed in mammalian tissues and is a major constituent of the milk fat globule membrane, that surrounds fat globules in milk.^6,7^ Xanthine oxidoreductase catalyzes the hydroxylation of hypoxanthine to xanthine and of xanthine to uric acid (UA)^6^ (Fig. 1). Xanthine oxidoreductase occurs in two interconvertible forms, xanthine dehydrogenase and xanthine oxidase. Xanthine dehydrogenase reduces the cofactor NAD, and xanthine oxidase prefers molecular oxygen (O_2_) as its electron acceptor. Both interconvertible forms, but particularly the xanthine oxidase form, produce a wide range of reactive oxygen and nitrogen species. As a result, the synthesis of numerous free radicals (superoxide (O_2_•−), hydrogen peroxide (H_2_O_2_), and antioxidant compounds, such as UA makes xanthine oxidoreductase an important protective regulator of the cellular redox potential.^8^

Until recently xanthine oxidase was thought to be a structural function protein in the milk fat globule membrane. Its function was thought to depend only on its protein structure, such as completing the process that leads milk lipid secretion, being independent of its enzymatic activity. However, Bjorck and Claesson proposed that xanthine oxidase-derived H_2_O_2_ exerts its antimicrobial effects by acting as a substrate for the lactoperoxidase system in bovine milk.^9^ It has been recently shown that the addition of 100 μM hypoxanthine (xanthine oxidase substrate) to breast milk promotes the production of H_2_O_2_.^10^ H_2_O_2_ has been well described chemically and its generation has been demonstrated to inactivate various microorganisms.^11^ A recent study has demonstrated that when human milk interacts with the infant saliva, the amount of H_2_O_2_ produced during nursing inhibits the growth of opportunistic pathogens, such as *Staphylococcus aureus* and *Salmonella* spp. The inhibitory metabolites produced by xanthine oxidase (e.g., H_2_O_2_) thus have the potential to regulate the oral microbiota of the infant.^5^ It has been recently demonstrated that the antibacterial activity of bovine milk against *S. aureus* is dependent on the hydroxylation of hypoxanthine by xanthine oxidase, leading to the production of H_2_O_2_ (unpublished data). The implications and biological properties of the antimicrobial enzyme xanthine oxidase towards infant health have not been well established, nor have the consequences of processing milk before consumption.

Non-human milks also contain xanthine oxidase and thus the enzymes are potentially important to the health of those consuming dairy products. Milk is heat treated prior to human consumption to ensure its microbial safety and to improve its shelf life.^12,13^ Thermal pasteurization is the method of choice by the dairy industry and requires specific time and temperature protocols. The choice of pasteurization conditions is dictated by both inactivation of microorganisms and shelf life extension. Accurate control of temperature has led to the use of rapid heating and cooling to enable effective microbial inactivation and better preservation of milk quality. Bovine milk can be pasteurized at high temperature short time (HTST), where milk is heated at 72 °C for at least 15 s^13^ or at higher temperatures for shorter times (ultra high temperature (UHT)), where milk is heated at 135−150 °C for 2−6 sec.^14^ Human milk banks still use the batch pasteurization (BP) process where milk is heated to 63 °C for 30 min.

The effects of the various heat treatments to which milk is commonly subjected to on xanthine oxidase activity is not fully understood and current research has so far focused on xanthine oxidase activity only. To the best of our knowledge there are no reports describing the effects of heat treatments on xanthine dehydrogenase. While xanthine oxidase activity has been undetected in infant formula and pasteurized human milk by some researchers,^5^ the effect of heat treatment is not clear^15–18^ (Table 1). Most of the previous research has used laboratory apparatus to simulate industrial heat treatments, making it difficult to compare or to translate the observed effects to a continuous pilot-scale or industrial pasteurization equipment.

**Table 1:** Comparative thermal stability results for xanthine oxidase activity

Sample	Processing conditions	Remaining xanthine oxidase activity (%)	References
Bovine milk	63 °C for 30 min	39	^18^
Bovine milk	70 °C for 15 s	84	^26^
Bovine milk	75 °C for 15 s	68	^26^
Bovine milk	73 °C for 7 min	Retains enzyme activity	^27^
Bovine milk	73 °C for 15 s	44	^18^
Bovine milk	70°C for 15 s^a^	103	^17^
Bovine milk	75 °C for 15 s^a^	100	^17^
Bovine milk	70 °C for 15 s^a^	110	^17^
Bovine milk	70 °C for 15 s^b^	95	^17^
Bovine milk	75 °C for 15 s^b^	92	^17^
Bovine milk	80 °C for 15 s^a^	27	^17^
Bovine milk	80 °C for 50 s	Retains enzyme activity	^27^
Bovine milk	62.7 °C for 30 min spray pasteurizer	38	^16^
Bovine milk	73.8 °C for 15 s mallory	58	^16^
Bovine milk	90.5 °C for 15 s mallory	0	^16^
Bovine milk	63 °C for 30 min^c^	92	This work
Bovine milk	72 °C for 15 s^c^	100	This work
Bovine milk	135 °C for 3 s^c^	4	This work

^a^The laboratory heat treatments were performed using a water jacketed stainless steel coil

^b^Pilot-scale experiments were performed using an APV Junior Paraflow plate heat exchanger

^c^Pilot scale experiments were performed in Advanced Milk Processing Laboratory (Davis, CA, USA), using a continuous ultra-high-temperature/high-temperature short time (UHT/HTST) pasteurizer (MicroThermics, Raleigh, NC, USA)

The objective of this study was to determine the effects of industrial milk processing conditions on the activity and biological functions of xanthine oxidase, xanthine dehydrogenase, and xanthine oxidoreductase using a continuous UHT/HTST pasteurizer. Milks were evaluated for the effects of HTST, UHT, and BP on: (a) kinetic parameters of xanthine oxidase, xanthine dehydrogenase, and xanthine oxidoreductase inactivation and storage stability (+4, −20, and −80 °C for a week) and (b) the ability of xanthine oxidase in BP, HTST, and UHT milks to inhibit the growth of *S. aureus*. Understanding the chemical reaction rates arising during food processing and storage and the mechanisms associated with those changes is important to selecting the best processing and storage conditions to preserve the activity of xanthine oxidoreductase.

## Results and discussions

### Xanthine oxidase, xanthine dehydrogenase, and xanthine oxidoreductase activities following heat treatments

The effects of BP, HTST, and UHT on the activities of xanthine oxidase, xanthine dehydrogenase, and xanthine oxidoreductase were assayed directly using 300 μM xanthine concentration. Raw milk was used as a control for all experiments and exhibited an average activity of 200.0 ± 12.3, 62.6 ± 8.5, and 263.2 ± 8.2 U/L for xanthine oxidase, xanthine dehydrogenase, and xanthine oxidoreductase, respectively. Our results are in agreement with the ones presented by Demott and Praepanitchai (1978). Despite differences in the methods used in both studies, similar values of xanthine oxidase activity were observed in both studies (208 vs. 200 U/L). In their study, xanthine oxidase activity was quantified by measuring the rate of formation of vanillic acid from vanillin, while in ours, xanthine oxidase activity was measured based on the rate of oxidation of xanthine to UA. Overall, there is a wide range of variation for bovine milk xanthine oxidase activity reported in the literature, which might be, in part, due to different reaction conditions used by different methods to quantify xanthine oxidase activity (temperature, pH, and reaction substrates). For example, Zikakis and Wooters (1980) reported an average xanthine oxidase activity (38 °C and pH 7.5) of 61.2 μL/O_2_ (40 U/L), while Cerbulis and Farrell (1976) reported xanthine oxidase activities (25 °C and pH 7.4) for raw bovine milk from 75.7 to 151 U/L by directly using 133 μM of xanthine concentration, which is in agreement with our study when similar concentration of xanthine (150 μM) is used. The observed differences could be also expected due to variations in milk associated with the sexual cycle of the cow, season, feeding regime, and breed.^19^

**Fig. 2:**
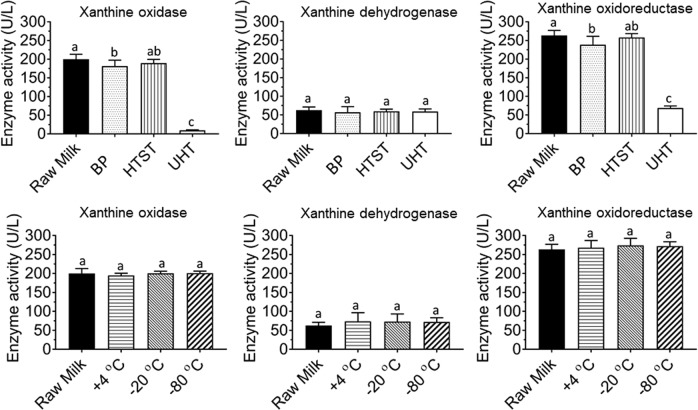
Effects of heat treatments^†^ and different storage conditions (+4, −20, −80 °C for a week) on xanthine oxidase, xanthine dehydrogenase, and xanthine oxidoreductase activities, respectively. Means with the same letter are not statistically different at *p* < 0.05. ^†^High-temperature short time (HTST; 72 °C for 15 s), batch pasteurization (BP; 62.5 °C for 30 min), and ultra pasteurization (UHT; 135 °C for 3 s)

In this study, HTST enabled complete retention of the activities of xanthine oxidase, xanthine dehydrogenase, and xanthine oxidoreductase (189.61 ± 9.17, 59.67 ± 9.36, and 257.92 ± 9.2 U/L, respectively) compared with raw milk (200.0 ± 12.3, 62.6 ± 8.5, and 263.2 ± 8.2 U/L, respectively) (Fig. 2). These results are in agreement with the ones reported by Griffiths (1986), who evaluated the thermal resistance of several indigenous enzymes in milk and concluded that xanthine oxidase was unaffected by heating at 75 or 70 °C for 15 s but lost 70% of its activity after being subjected to 80 °C for 15 s.

However, our results differ from the ones reported by Greenbank and Pallansch (1962), Andrews et al. (1987), and Sharma et al. (2014) (Table 1). The variations observed for xanthine oxidase activities in these studies (15–56% reduction at 70–75 °C for 15 s) could be attributed to different temperature–time combinations used and the type of heating system used (sealed thin glass capillary tube, water bath mounted on a computer-controlled ceramic hot plate, or a Mallory-type heat exchanger).

Similar to HTST, BP enabled a high preservation of xanthine oxidoreductase (283.49 ± 9.36 vs. 263.2 ± 8.2 U/L), xanthine oxidase (181.3 ± 15.21 vs. 200.0 ± 12.3 U/L), and xanthine dehydrogenase (57.13 ± 9.31 vs. 62.6 ± 8.5 U/L) activities, compared to raw milk. Overall, the use of BP reduced xanthine oxidoreductase and xanthine oxidase activities by 10%, respectively (*p* < 0.05). These results differ from the ones reported by Sharma et al. (2014) and Greenbank and Pallansch (1962), in which the use of BP led to a 60% decrease in the activity of xanthine oxidase. As previously described, the observed differences in xanthine oxidase activities are likely due to the different types of processing systems used and different time–temperature combinations.

The use of UHT significantly reduced xanthine oxidoreductase (68.68 ± 5.67 U/L), Xanthine Oxidase (9.47 ± 1.68 U/L) activities compared with raw milk (*p* < 0.05). Xanthine oxidoreductase and xanthine oxidase activities decreased by 74% and 95%, respectively, when milk was subjected to the UHT process (*p* < 0.05). These results are in good agreement with trend observed in previous studies where higher temperatures (up to 80–90 °C) resulted in higher inactivation rates of xanthine oxidase (Table 1). In general, xanthine dehydrogenase activity was not reduced by any of the heat treatments applied compared to the control (raw milk).

### Xanthine oxidase, xanthine dehydrogenase, and xanthine oxidoreductase activities following different storage conditions (+4, −20, −80 °C for a week)

The effects of different storage conditions on raw milk xanthine oxidase, xanthine dehydrogenase, and xanthine oxidoreductase activities were evaluated by storing raw milk at +4, −20, and −80 °C for a week. The activities of xanthine oxidase and xanthine oxidoreductase stored at +4, −20, and −80 °C and fresh milk are shown in Fig. 2. No statistically significant changes were observed for the activities of xanthine oxidase and xanthine oxidoreductase when raw milk was stored at +4, −20, and −80 °C, compared with fresh raw milk. These results demonstrate that any of the storage conditions evaluated (+4, −20, and −80 °C) could be used for a short-term storage of one week. Our results are in agreement with the ones reported by Demott and Praepanitchai (1978), where average xanthine oxidase activity of raw milk remained constant after 96 h of storage at 4 °C (208 vs. 200 U/L). Similar finding was observed by Cerbulis and Farrell (1977), in which the storage of milk samples at 2 °C for 6 days had no detrimental effect on xanthine oxidase activity.

### Kinetic properties of raw and heat-treated bovine milk xanthine oxidase

**Fig. 3:**
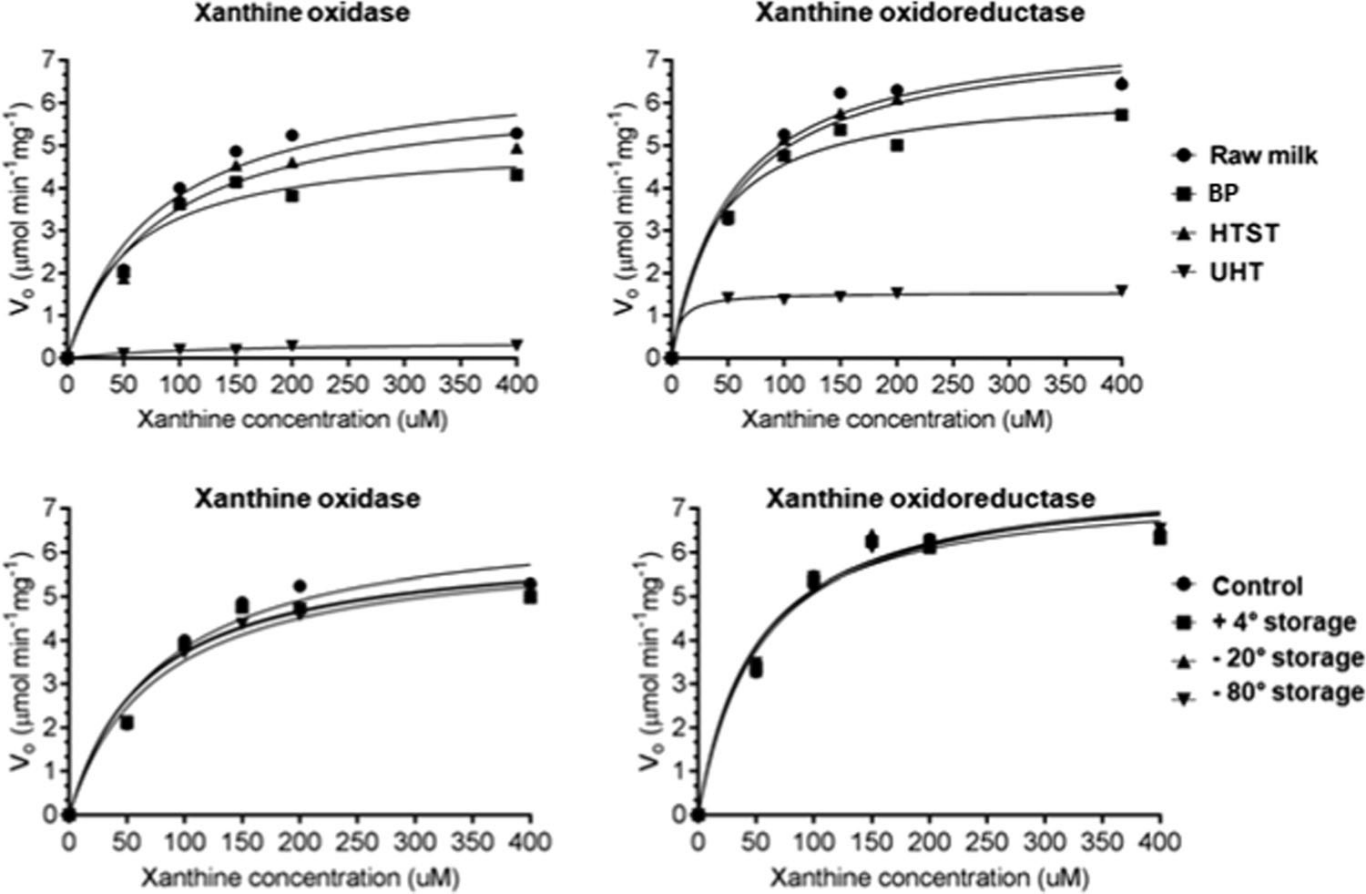
Kinetic parameters (*K*_m_, *V*_max_, *D*- and *Z*-values) of xanthine oxidase and xanthine oxidoreductase in raw, heat treated^†^, and a week stored milk at +4, −20, −80 °C. Michaelis–Menten plot of raw and heat treated^†^ and a week stored milk at +4, −20, −80 °C bovine milk xanthine oxidase and xanthine oxidoreductase. ^†^High-temperature short time (HTST; 72 °C for 15 s), batch pasteurization (BP; 62.5 °C for 30 min), and ultra pasteurization (UHT; 135 °C for 3 s)

In order to understand the effects of industrial heat treatments on xanthine oxidase activity, the Michalis-Menten parameters (*K*_m_—which denotes the affinity of the enzyme to the substrate and *V*_m_—the maximum velocity of the reaction) were evaluated using Graph Prism 6.0 software with non-linear plotting techniques.^20,21^ The kinetics of xanthine oxidase and xanthine oxidoreductase in raw, heat treated, and one week-stored milks (+4, −20, and −80 °C) were determined using various concentrations of xanthine (0, 50, 100, 150, 200, 400 Μm) for 6 min reaction at 37 °C. The reaction rate for each substrate concentration was determined from first 6 min, and initial rates of reaction were therefore calculated from Eq. (1) in the “Methods” section. The assumption that [*S*]»[*E*] is not valid after 6 min.^19^ Therefore, the substrate concentration becomes limited and the reaction rate slows after 6 min. Table 2 presents the kinetic parameters (*V*_max_, *K*_m_) obtained by fitting the data (Fig. 3) to the Michaelis–Menten model. The direct plot was created by plotting the reaction rate (*v*) against the initial substrate concentration (*S*). *K*_m_ values of 79.14, 55.24, 79.18, 132.7, 68.58, 67.58, and 85.88 μM were determined for fresh raw, BP, HTST, UHT and +4, −20, and −80 °C stored milk xanthine oxidase, respectively. *K*_m_ values of 53.99, 39.52, 56.46, 47.12, 51.74, and 49.23 were determined for fresh raw, BP, HTST and +4, −20, and −80 °C stored milk xanthine oxidoreductase, respectively (Table 2). *K*_m_ refers to the minimum substrate concentration that yields half of *V*_max_. The higher the *K*_m_, the lower the affinity of the enzyme to its substrate. The use of HTST did not alter the xanthine oxidase affinity to its substrate compared with raw milk (79.1 vs. 79.2 μM). However, a significant increase in the *K*_m_ value of the enzyme after the UHT treatment was observed compared with raw milk (132 vs. 79.1 μM), which indicates the effect of the UHT treatment on reducing the ability of xanthine oxidase to bind to its substrate.

**Table 2:** Summary of kinetic parameters (*K*_m_, *V*_max_, *D*- and *Z*-values) of xanthine oxidase and xanthine oxidoreductase in raw, heat treated, and a week stored milk at +4, −20, −80 °C

	Raw milk	BP	HTST	UHT	Raw milk +4 °C storage	Raw milk −20 °C storage	Raw milk −80 °C storage
*Xanthine oxidase*							
*K*_m_ (μM)	79.14	55.24	79.18	132.7	68.58	67.58	85.88
*V*_max_	6.85	5.115	6.31	0.40	6.23	6.26	6.55
*Xanthine oxidoreductase*							
*K*_m_ (μM)	53.99	39.52	56.46	1.54	47.12	51.74	49.23
*V*_max_	7.80	6.351	7.68	1.542	7.513	7.82	7.70
*Xanthine oxidase*							
*k* × 10^−5^ (s^−1^)	5.45	357.41	101676.48				
*D*-values (s)	42215.49	644.23	2.26				
*R*^2^		0.89	0.89	0.89			
*Z* (°C)		19.30					
*Xanthine oxidoreductase*							
*k* × 10^−5^ (s^−1^)	5.48	136.30	44784.23				
*D*-values (s)	41958.37	1689.24	5.14				
*R*^2^		0.94	0.94	0.94			
*Z* (°C)		20.40

Figure 3 shows the curves obtained for the velocity of xanthine conversion by the native, heat treated, and stored enzymes, measured at different substrate concentrations. *V*_max_ values of 6.85, 5.11, 6.31, 0.40, 6.23, 6.26, and 6.55 μmol/min/mg were determined for fresh raw, BP, HTST, UHT, and +4, −20, and −80 °C a week stored milks xanthine oxidase, respectively. *V*_max_ values ranging from 7.80, 6.35, 7.68, 1.54, 7.51, 7.82, and 7.70 μmol/min/mg were determined for fresh raw, BP, HTST, UHT, and +4, −20, and −80 °C stored milks xanthine oxidoreductase, respectively. These results indicate that the maximum rate of reaction is lower in UHT treatment (1.54 μmol/min/mg) compared to mild heat treatments (BP and HTST, 6.35–7.68 μmol/min/mg) and different short-time storage conditions (7.5–7.8 μmol/min/mg). In general, UHT inactivated xanthine oxidase. The high *K*_m_ value and the lower *V*_max_ observed for the UHT-treated milk indicates that xanthine oxidase affinity to its substrate was reduced and a higher concentration of substrate would be needed to achieve its maximum catalytic rate (*V*_max_), which was reduced by 95%. In contrast, HTST and raw milk had similar *V*_max_ and *K*_m_, suggesting that HTST milk processed by commercial scale equipment retains xanthine oxidase activity.

**Fig. 4:**
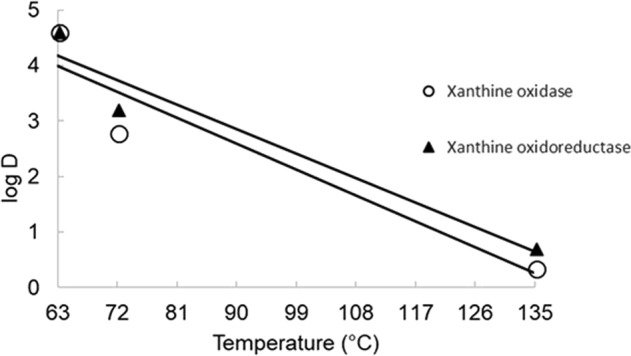
Effects of temperature on *D* values for the inactivation of bovine milk xanthine oxidase and xanthine oxidoreductase. Triangle and circle indicate xanthine oxidase and xanthine oxidoreductase, respectively

*D*-values (time for 90% reduction of enzymatic activity) for xanthine oxidase are shown in Table 2 and Fig. 4. *D*-values decreased at increasing temperature within 63 and 135 °C. These results demonstrate that xanthine oxidase activity is slowly inactivated below 72 °C, with an increasing rate of inactivation being observed at 135 °C. Processing milk at 63 °C required 703, 751, and 700 min to reduce xanthine oxidase, xanthine dehydrogenase, and xanthine oxidoreductase by 90%, respectively (Table 2). However, at 72 °C, only 10, 8, and 28 min of treatment resulted in the same degree of inactivation. At 135 °C, 2.26, 123.03, and 5.14 s were necessary to reduce the xanthine oxidase, xanthine dehydrogenase, and xanthine oxidoreductase activity by 90%. Higher *D*-values for inactivation of enzyme suggest higher resistance to thermal treatment. The *Z*-value was calculated and found to be 20 °C for both xanthine oxidase and xanthine oxidoreductase, for the range of temperatures studied (63, 72, and 135 °C). In general, low *Z*-values mean greater sensitivity to increases in temperature and higher *Z* values mean more sensitivity to the duration of treatment.^22^ *D*-values of 224.7, 29.7, 5.06, 1.39 min were reported by Griffiths (1986) for the thermoresistance of bovine milk xanthine oxidase at 65, 70, 75 and 80 °C for 15 s, respectively. The results obtained in our study with the use of a continuous pilot-scale pasteurizer (72 °C for 15 s and 135°C for 3 s; *D*-values of 10.7 and 0.03 min, respectively) are closer to the ones presented by Griffiths (1986) at 70° and 75 °C (*D*-values of 29.7–5.06 min). Differences in temperatures and holding times (30 min for BP vs. 15 s for the continuous process) and the different pilot-scale pasteurizer systems used (ours being a continuous tube heat exchanger pasteurizer with pre-heating and theirs being a plate heat exchanger with no pre heat) are interpreted as possible causes of variations in *D* values.

### Effects of heat treatments on xanthine oxidase antimicrobial activity

**Fig. 5:**
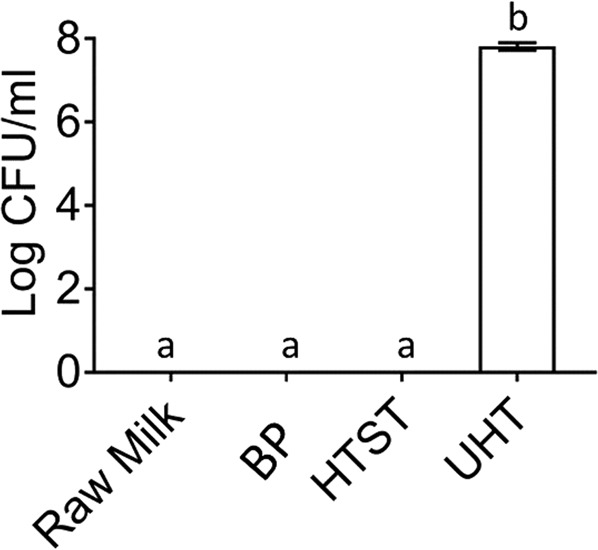
Effects of heat treatments^†^ of bovine milk xanthine oxidase on bacterial counts (CFU/mL). Bacteria were incubated with raw or heat treated^†^ milks and xanthine. Means with the same letter are not statistically different (*p* < 0.05). ^†^High temperature short time (HTST; 72 °C for 15 s), batch pasteurization (BP; 62.5 °C for 30 min), and ultra pasteurization (UHT; 135 °C for 3 s)

In addition to UA, xanthine oxidase also generates H_2_O_2_ as a product (Fig. 1). Recent studies have highlighted that the biological functions of xanthine oxidase are associated with the production of H_2_O_2_.^5,8^ The effects of heat treatments with different intensities (BP, 63 °C for 30 min; HTST, 72 °C for 15 s; and UHT, 135 °C for 3 s) were evaluated regarding the antibacterial activity of bovine milk xanthine oxidase against *S. aureus* (Fig. 5). To evaluate the potential antibacterial activity of xanthine oxidase after subjected to the above heat treatments, xanthine oxidase in growth-inhibition assays of *S. aureus* were performed. In this assay, the viability of *S. aureus* in different media (raw and heat-treated milks) in the presence of 200 μM of xanthine was evaluated by explicit measurement. Enzyme activity might have potentially generated 200 μM H_2_O_2_, which resulted in inactivation of the bacterial counts (CFU/mL) after 24 h of growth. As detailed in Fig. 5, BP and HTST milk xanthine oxidase activity inhibited the growth of *S. aureus*. When xanthine oxidase activity was diminished by the UHT treatment (135 °C for 3 s), the counts of *S. aureus* were unaffected by the presence of milk (*p* < 0.0001).

Collectively, for all samples, xanthine oxidase activity correlated directly with the antimicrobial properties of xanthine oxidase (Fig. 5). The reduced xanthine oxidase activity (95% inactivation) for UHT milk likely reduced the production of H_2_O_2_ thus lowering its antibacterial activity as compared to fresh raw, BP, and HTST milks. The effects of heat treatments on xanthine oxidase antimicrobial activity at pH 7.5 and at 37 °C in milk were previously unknown.

The effects of several heat treatments on the biological activities of xanthine oxidase, xanthine dehydrogenase, and xanthine oxidoreductase were evaluated. Xanthine oxidase activity was maintained by HTST, with partial (8%) and nearly complete inactivation (95%) being observed for BP and UHT milks, respectively. While the use of UHT resulted in complete enzyme inactivation, HTST at 75 °C for 15 s or BP at 63 °C for 30 min maintained the catalytic and antimicrobial properties of this enzyme. HTST and raw milk xanthine oxidase had similar *V*_max_ and *K*_m_, suggesting that HTST milk processed by industrial processing technologies maintains xanthine oxidase activity. *D*-, *z*- and *k*-values indicate that xanthine oxidase is stable to the HTST conditions tested, maintaining its biological activity at this processing condition.

## Methods

### Milk samples

Fresh raw bovine milk was obtained from the UC Davis dairy farm (Davis, CA, USA). Raw milk was heat-treated at the Advanced Milk Processing Laboratory (Davis, CA, USA) using an indirect UHT/HTST Lab 25 EHV Hybrid w/PLC Touchscreen Control (MicroThermics, Raleigh, NC, USA) tubular continuous pasteurizer. Milk samples were preheated at 65 °C and subsequently subjected to 72 °C for 15 s (high temperature short time (HTST)) and to 135 °C for 3 s (UHT). BP (63 °C for 30 min) was performed using a 10 L jacketed reactor (Chemglass, Life Sciences, Vineland, NJ). Each heat treatment was performed in triplicate, with a new batch of raw milk being used for each replicate. Raw and heat-treated samples were stored at −80 °C until use. Raw and heat-treated milk samples were analyzed within 1 day of milking and processing. Samples were analyzed after one-week storage at +4, −20, and −80 °C.

### Enzyme assays and kinetic parameters

#### Xanthine oxidase, xanthine dehydrogenase, and xanthine oxidoreductase activities

Activity of xanthine oxidase, xanthine dehydrogenase, and xanthine oxidoreductase were determined by spectrophotometry (Thermo Scientific, GENESYS 10S UV–Vis, USA). The rate of oxidation of xanthine to UA was measured at 290 nm for 6 min reaction according to refs^19,23^ Enzyme activity measurements were performed at 37 °C and pH 7.5 in a Tris–HCl buffer solution. 300 µM concentration of xanthine (final concentration of 100 µM xanthine) was directly added for the quantification of xanthine oxidase and 300 µM xanthine and 1.5 mM NAD^+^ (final concentration of 100 µM xanthine and 500 µM NAD^+^) were added for the quantification of xanthine oxidoreductase. Assay tubes (total volume 3 mL) were prepared with 1 mL of 7.5 in a Tris–HCl buffer and 0.2 mL milk sample. The reaction was stopped by the addition of 20% trichloroacetic acid. The dehydrogenase activity of the enzyme preparation was determined by subtracting the xanthine oxidase value from xanthine oxidoreductase value. With xanthine as a reducing substrate, UA production was monitored as described above by using a molar absorption coefficient (*ε*) of 1.22 mM^−1^ cm^−1^. Measurements were performed at least in triplicate, and error bars represent one standard deviation.

### Kinetic parameters

Steady-state kinetic experiments were carried out to determine the rate of heat-induced effects on xanthine oxidoreductase activity. Using the Michaelis–Menten model, the velocity of the reaction can be obtained from the initial linear decrease of the substrate concentration or from the increase of the product concentration. *K*_m_, the substrate concentration when the reaction reaches half of *V*_max_, can also be calculated from the obtained curve.^20^ For xanthine oxidase activity measurements, final concentrations of its reducing substrate xanthine (0, 50, 100, 150, 200, and 400 μM) were added and the production of UA was measured for 6 min reaction at 37 °C.^19^ For xanthine oxidoreductase activity, both final concentration of xanthine (0, 50, 100, 150, 200, and 400 μM) and NAD^+^ (500 μM) were used as reducing substrates and their consumption was measured at 290 nm. Accordingly, the reaction rate (*v*) of xanthine oxidase, xanthine dehydrogenase, and xanthine oxidoreductase, the concentration of a substrate [*S*], can be described by the Michaelis–Menten equation (Eq. 1):

1 $$v = \frac{{k_{\mathrm {c}}\left[ {E_{\mathrm{o}}} \right]\left[ S \right]}}{{S + K_{\mathrm {m}}}}$$

where [*E*_o_] is the concentration of enzyme, and *k*_c_ and *K*_m_ are the catalytic constant of hydrolysis and the Michaelis constant, respectively.

A first-order reaction can be used to describe the heat inactivation of xanthine oxidase, xanthine dehydrogenase, and xanthine oxidoreductase at 72 °C for 15 s (HTST), 135 °C for 3 sc (UHT), and at 63 °C for 30 min (BP). At a given temperature, the rate of inactivation of xanthine oxidoreductase can be described by (Eq. 2)

2 $$k = \frac{{{\mathrm {ln}}\left( {A_{0}/A} \right)}}{t}$$

where *k* is the reaction constant (s^−1^), *A*_0_ is the initial activity at *t* = 0, and *A* is the residual activity at time *t*, after the heat treatment. *A*/*A*_0_ is the residual activity of xanthine oxidase and xanthine oxidoreductase after time of heating (s) at a given temperature. A more practical form to express enzyme inactivation is to determine the decimal reduction time (*D*-value), which represents the heat resistance of the enzyme at a given temperature.^24^ The *D*-value, the time needed to reduce the initial enzyme activity by 90% (one log cycle), was calculated according to Eq. (3):

3 $$D = \frac{{{\mathrm {ln}}10}}{k}$$

To describe the temperature dependence of xanthine oxidase and xanthine oxidoreductase inactivation in the context of microbial inactivation, the *z*-value (the temperature rise needed to reduce the *D*-value by one log cycle (90%), was obtained by regression analysis of the line obtained by plotting the logarithm of *D*-values against the corresponding temperatures.^25^ All measurements were performed at least in triplicate.

### Inhibition of bacterial growth by xanthine oxidase

While enzyme activity reduction will lead to a reduction in the antimicrobial properties of xanthine oxidase, it is important to determine the magnitude of activity loss that would still maintain the inhibitory functions of this enzyme. In order to understand the effects of heat treatments on inhibition of bacterial growth by xanthine oxidase, *S. aureus* (*ATCC 29213*), was used as a model microorganism.^5^ To this end, stocks of bacteria species *S. aureus* were diluted in sterile phosphate-buffered saline. Bacteria were incubated with raw, BP, HTST, and UHT milks and milks+xanthine. Each microtiter well contained 50 μL of xanthine solutions with a final concentration of 200 μM, 25 μL diluted milk, 25 μL bacteria with a final concentration of 100 CFU/mL bacteria, in a total volume of 100 μL (in triplicate). The 96-well plates were incubated for 24 h at 37 °C. Serial dilutions were made and aliquots were then inoculated onto agar plates, incubated and viable CFU counted.^5^ The final concentration of xanthine was 200 μM, which theoretically can produce 200 μM H_2_O_2_. The assays were performed in triplicate and error bars represent one standard deviation.

### Statistics

Michaelis–Menten kinetic model parameters were analyzed by Graph Prism 7.0 software with non-linear plotting techniques.^20,21^ One-way ANOVA by Graph Prism 7.0 software was used for evaluating the effects of the different heat treatments on xanthine oxidase, xanthine dehydrogenase, and xanthine oxidoreductase activities and biological activities. Multiple comparison of the least-square means was made by Tukey’s adjustment with level of significance set as *p* < 0.05.

### Reporting Summary

Further information on research design is available in the Nature Research Reporting Summary linked to this article.

## Supplementary information

Reporting Summary

## Data Availability

The datasets generated during and/or analyzed during the current study are available from the corresponding author on reasonable request.

## Supplementary information

**Supplementary information** accompanies the paper on the *npj Science of Food* website (10.1038/s41538-019-0046-8).
